# Association between job strain and working life expectancy: a longitudinal study of older people in Sweden

**DOI:** 10.1093/eurpub/ckae186

**Published:** 2024-12-12

**Authors:** Holendro Singh Chungkham, Robin Högnäs, Kristina Alexanderson, Paola Zaninotto, Kristin Farrants, Martin Hyde, Linda L Magnusson Hanson, Jenny Head, Reiner Rugulies, Ann Dyreborg Larsen, Anushiya Vanajan, Sari Stenholm, Hugo Westerlund

**Affiliations:** Psychobiology and Epidemiology Division, Department of Psychology, Stockholm University, Stockholm, Sweden; Department of Epidemiology and Public Health, University College London, London, United Kingdom; Psychobiology and Epidemiology Division, Department of Psychology, Stockholm University, Stockholm, Sweden; Division of Insurance Medicine, Karolinska Institute, Stockholm, Sweden; Department of Epidemiology and Public Health, University College London, London, United Kingdom; Division of Insurance Medicine, Karolinska Institute, Stockholm, Sweden; School of Business, University of Leicester, Leicester, United Kingdom; Psychobiology and Epidemiology Division, Department of Psychology, Stockholm University, Stockholm, Sweden; Department of Epidemiology and Public Health, University College London, London, United Kingdom; National Research Centre for the Working Environment, Copenhagen, Denmark; Section of Epidemiology, Department of Public Health, University of Copenhagen, Copenhagen, Denmark; National Research Centre for the Working Environment, Copenhagen, Denmark; Department of Sociology, Faculty of Social Sciences, Vrije University Amsterdam, Amsterdam, The Netherlands; Department of Public Health, University of Turku and Turku University Hospital, Turku, Finland; Centre for Population Health Research, University of Turku and Turku University Hospital, Turku, Finland; Research Services, Turku University Hospital and University of Turku, Turku, Finland; Psychobiology and Epidemiology Division, Department of Psychology, Stockholm University, Stockholm, Sweden

## Abstract

Many European countries have increased retirement ages to address the challenge of population ageing. However, job strain which is the combination of high job demands and low job control may be an obstacle to extending the working lives of older workers. Job strain is associated with poor health and early work exit among older workers, but less is known about whether job strain impacts working life expectancy (WLE)—an increasingly employed summary measure capturing the length of working lives. This study aims to fill this gap in the literature. The sample included *n* = 13 225 individuals aged 50 years or older at baseline providing 53 004 persons-observations from the Swedish Longitudinal Occupational Survey of Health in 2008 through 2020. We used continuous time multi-state Markov models to assess the average number of years people may be expected to work beyond age 50 years by job strain, and stratified by sex, occupational class, and level of education. Job strain was associated with a significantly shorter WLE (by about 6 months to a year) among those who experienced job strain compared to those who did not experience job strain. Our findings suggest that job strain may play a role in shortening the working lives of older people. The findings further suggest that if older workers are to remain in the labor market for longer periods, this may require improvements of psychosocial working conditions.

## Introduction

Population ageing has led many high-income countries to increase retirement ages, and labor market participation rates among older workers have increased in all OECD countries since 2000 [1]. In addition, new ways of working in later life are emerging in several countries; for instance, a growing number of older people have bridge jobs, have “unretired,” or combine work and retirement [1, 2].

While the economic rationale for these policies is understandable there are concerns that extending working lives may exacerbate inequalities in later life. Older workers who experience unfavorable working conditions and remain in these jobs may be exposed to these poor working conditions for longer periods. These situations may negatively impact their health and well-being [3–10]. Moreover, older workers with unfavorable working conditions may find themselves unable to meet the “new normal” of working longer—such conditions may lead to unemployment, long-term sickness absence, disability pension, or early retirement, which could have important financial consequences [11].

An important working condition that may impact the working lives of older people is job strain. The concept of job strain is based on Karasek’s [12] job demand-control model, and is defined as the combination of high job demands and low job control [13]. Prior research suggests that job strain negatively impacts workers’ health and work ability [14]. For example, job strain is associated with a range of health conditions, e.g. coronary heart disease [10], ischemic stroke [8], and depression [9]. Job strain among older workers may also increase early labor market exit. Indeed, research has shown that job strain is associated with increased preferences for and expectations of early retirement [15]. Several studies have further found that job strain increases the likelihood of taking early retirement and retiring due to disability [16, 17]. Taken together, prior research shows that job strain is a health risk factor and an adverse working condition linked to early labor market exit. Job strain is a potentially modifiable factor which could be addressed through policy efforts and organizational practices. A reduction in job strain may help retain older workers in the labor market, who then pay into social security systems [18] over a longer period while also increasing pension savings.

### The current study

Prior research on job strain and work later in life has primarily focused on early retirement and the timing of retirement. However, research suggests that in many Western countries, transitioning into retirement is no longer a one-step process, rather a heterogeneous process that can include many push and pull factors that often vary by social situation [19]. For example, it is increasingly common for older workers to have postretirement jobs, bridge jobs, phased retirement, and negotiate flexible work arrangements [2, 20, 21]. Thus, measures of retirement timing such as the average age of labor market exit may not sufficiently capture the diversity of working lives, particularly transitions between working states, at older ages. One example of this is when one retires but also works while technically being “retired”. The complexity in transitions between work and retirement among older workers is better captured using working life expectancy (WLE). WLE can be defined as the average expected number of years a person may be expected to work beyond a given age [22]. WLE is a more holistic measure of the total contribution of work done [22–26] as it includes the time in work, e.g. when someone retires and returns to work part-time. Very few studies have examined how WLE relates to working conditions. Unfavorable working conditions including high physical demands (Pedersen2020, Schram 2021, Schram 2022) and psychosocial working conditions (Schram 2022) such as job demands and lack of autonomy have been found to be associated with shorter WLEs; however, little is known about the effects of job strain on the WLEs of older workers.

The current study examines the role of job strain in WLE among older workers in Sweden. Over the last two decades, Sweden has not only increased retirement ages, but access to disability pensions has also been tightened. These policy shifts thus encourage work into old age with few considerations for the working conditions that may impede longer working lives. Therefore, the findings from the current study have implications for both the well-being and working conditions of older workers and for policies to extend working lives.

## Methods

### Data

Data are from the Swedish Longitudinal Occupational Survey of Health (SLOSH) study, focusing on work life, health, and welfare [27]. SLOSH initially included 9214 gainfully employed workers, aged between 16 and 64 years, from the 2003 Swedish Work Environment Survey (SWES). These individuals were firstly selected through stratified random sampling from the working-age population for the Labor Force Survey (LFS), and secondly by work status for SWES, and are thereby approximately representative of the Swedish working population. After responding to LFS interviews and SWES questionnaire (only for individuals in paid work in 2003), they were invited to respond to SLOSH follow-up questionnaires between 2006 and 2022, every 2 years. Refresher samples from SWES 2005–2011 were also added between 2008 and 2014. From 2014 to 2020, the total SLOSH population then amounted to 40 877 individuals. SLOSH data are linked to administrative register data, from which measures of occupational class, educational level, and year of death were drawn. For a more detailed description of the SLOSH study design, see Magnusson Hanson *et al.* [27] Further information on a number of respondents to SWES 2003–2011 and SLOSH 2006–2020 are shown in Supplementary Tables S3 and S4.

The current study is focused on the WLE of older workers and thus is focused on those aged 50 (to capture the preretirement period) to 75 years (the oldest age of those observed to be in work). Because of the study design, we do not have a fixed baseline in the study. For an individual who is age 50 years and in work in any of the waves of survey 2008, 2010, 2012, 2014, 2016, 2018, or 2020 for the first time is the baseline. Those with missing on either job strain or occupation or education at baseline were excluded. The inclusion and exclusion criteria of sample observations and person-observations are shown in the flowchart (see Supplementary File).

The primary analyses controlling for sex were based on an analytical sample of *n *=* *13 225 respondents which provides *n *=* *53 004 person-observations. In order to supplement the primary analyses controlling for occupational class and educational level, we again used two analytic samples in the analyses (see the flowchart in Supplementary Fig. S1). The first analytic sample included *n *=* *12 832 respondents which provides *n *=* *51 852 person-observations. Models were cross-classified by sex, occupational class, and job strain. All respondents were aged over 50 years, were working at baseline (between 2008 and 2020), and were nonmissing on job strain and the covariates (such as sex and occupation) at baseline. The second analytic sample included *n *=* *12 876 individuals which provides *n *=* *52 100 person-observations and the same age criterion, information on covariates (sex, education), and the exposure, job strain. Follow-up periods were between 2 and 12 years between 2008 and 2020 with *n *=* *2666, *n *=* *2741, *n *=* *3492, *n *=* *1252, *n *=* *1142, and *n *=* *1539 individuals followed for 2, 3, 4, 5, 6, and 7 follow-up periods, respectively.

### Measurements

#### Exposure

Based on Karasek’s job demand-control model [13], job strain was measured using self-reports about job demands and job control. The job demands consist of four items, e.g.: “Do you have to work very fast?”, and, job control consists of five items, e.g. “Do you have a choice in deciding what you do at work?”. Response items, measured on a Likert scale (1: Yes often to 4: No, hardly ever/never) were averaged; those whose scores were above the median were coded as having experienced job strain [8]. All scores below the median were coded as not experiencing job strain. The factor structure and its measurement invariance have been validated in SLOSH [28]. This measure is consistent with prior specifications of job strain [12, 13].

#### Outcome

**Figure 1.**
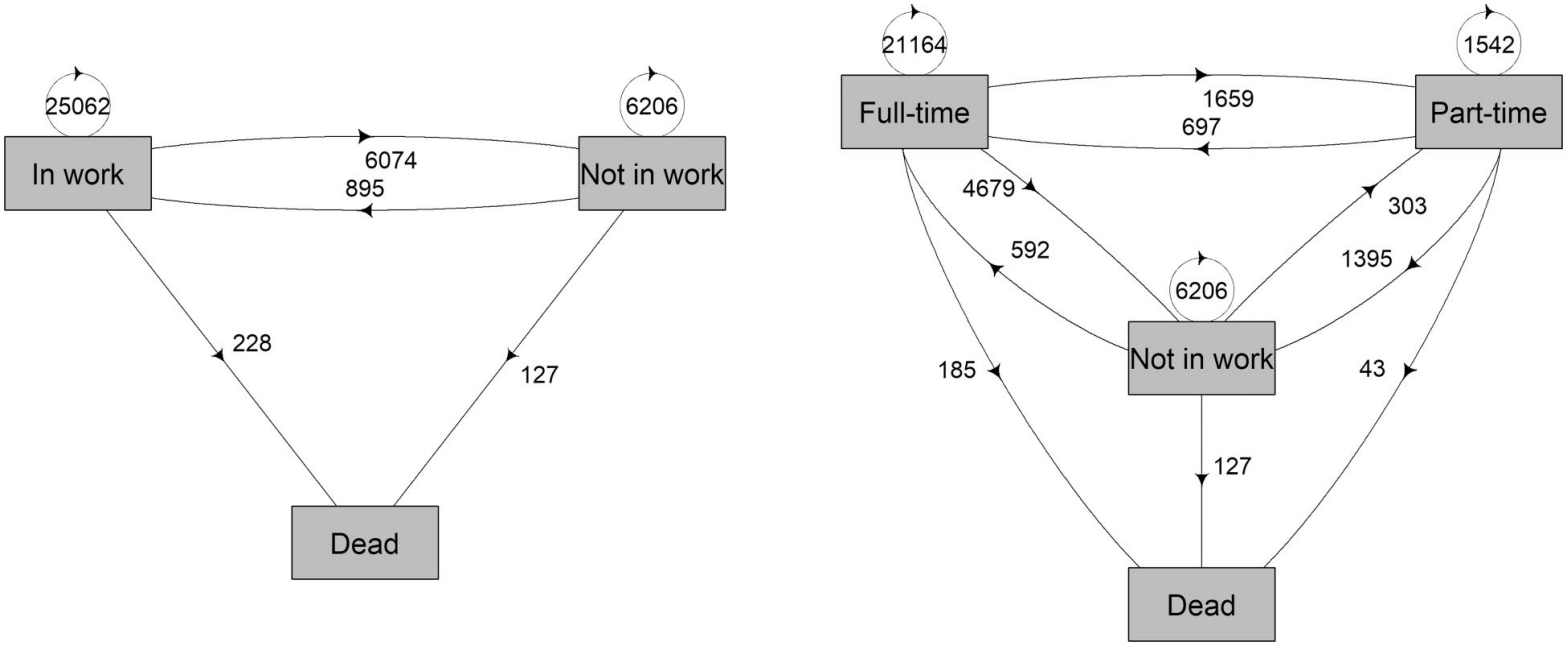
The three- and four-state models to estimate working life expectancies. The transitions are indicated by the arrows, and the figures indicate observed numbers of transitions between and within the non-absorbing states.

The outcome variable was a composite measure of individuals’ work and death statuses over time. Full-time work was defined as 30 work hours or more per week, part-time as 10–29 work hours per week, and, since we are interested primarily in how much older workers gainfully contribute to pension and healthcare systems, not in paid work was defined as fewer than 10 work hours per week. There were also few people who worked fewer than 10 h and this limited time in work also disqualifies people for some public benefits (e.g. unemployment insurance). Dates of death were linked to SLOSH from the Cause of Death Register. We estimated three- and four-state models. The four-state included: 1 = working full-time, 2 = working part-time, and 3 = not in work, and the absorbing state 4 = death. To estimate the total WLE, we collapsed full- and part-time work into one state, “working” to fit the three-state model. Figure 1 illustrates these multistate models; arrows indicate a possible transition between each state.

#### Covariates

The primary results were based on multi-state models with sex as the only covariate and, job strain as the exposure of interest. To supplement the findings, we ran two additional multi-state models with occupational social class, and educational level as co-variates, in addition to sex. Previous studies have shown that sex, occupational social class, and education are associated with WLE [25, 29]. The first set of three- and four-state models included sex and occupational class, and the second set included sex and educational level. Sex was dichotomized as male or female. Occupation was measured using self-reported job title and coded according to the Swedish socioeconomic classification [30]. Following previous research [27, 31], we collapsed occupation into three levels: Routine (e.g. manual workers), intermediate (e.g. assistant, and intermediate nonmanual employees), and professional (e.g. executives, professionals, and other high-level nonmanual employees). Educational level was measured using three categories: Highly educated (university level or higher), moderately educated (secondary school or high school), and lower educated (compulsory school; ninth grade or lower).

### Analytic approach

We employed multi-state models that were rooted in survival analysis, and have an underlying stochastic process, to estimate the transition probabilities between work states and death (the absorbing state). These models accounted for changes in the work statuses among people whose life cycles were exposed to contemporaneous trends in the labor market and mortality. Multistate models allow for transitions in and out of paid work among older workers, unlike methods that rely on prevalence rates such as the Sullivan method [32]. Moreover, multistate models can be used to examine the effects of various factors on older workers’ transitions between different states, and how long older workers remained in a specific state irrespective of their initial state. Thus, this study effectively reports marginal working life expectancies.

Hazard rates or instantaneous transition probabilities were estimated using the package *msm* [33] in *R* [34]. The model considers age as a continuous dependent variable, allowing the transition probabilities between states to increase or decrease log-linearly following a Gompertz function. The Gompertz function assumes a constant piecewise approximation that: (i) is conditioned on age, and (ii) where the instantaneous probability of transitioning between two states is constant within each interval of age [35]. The *msm* package only allows the calculation of expectancies based on an age-constant exponential function [35]; therefore, we used the *elect* package [35] to estimate working life expectancies, again, based on the Gompertz function. We calculated 95% confidence intervals (CI) for the estimates of WLE from 500 repeated simulations based on asymptotic properties of the maximum likelihood estimator for applied multi-state models [35, 36]. Both three- and four-state models take advantage of the censoring facility available in the *msm* package. Since we do not know the exact timing of transitions between the 2 years between data waves, nonabsorbing states were censored in the interval.

## Results

Figure 1 illustrates the three- and four-state multistate Markov models that were used to estimate transitions between states and subsequently WLEs. Notably, there were 895 transitions from “not in work” to “in work,” 228 transitions from “in work” to “death,” and 127 transitions from “not in work” to “death.” Since we divided working into “full-time” and “part-time,” there were 185 transitions from full-time work to death and 43 transitions from part-time work to death. There were 6074 transitions from “in work” to “not in work.” Lastly, 25 062 participants made no transitions out of being “in work” during the study period.

Table 1 shows the distribution of demographic variables for individuals included in the analysis. Women have a slightly higher percentage than men in the study sample with similar mean ages. Both men and, women work full-time than part-time with, higher percentage of women working as part-time than men. The results show that 21.2% of women experienced job strain compared to 15.7% of men. Men are more likely to be in the professional occupational social class than women, but women are more likely to have a higher education.

**Table 1.** Distribution of demographic characteristics of individuals at entry (SLOSH, 2008–2020)

	Sex	
Covariates	Men (*n* = 5862)	Women (*n* = 7363)
Age		
Mean (SD)	56.7 (5.2)	56.1 (4.9)
Work status, %		
Full-time	92.9	87.5
Part-time	7.1	12.5
Occupational class, %		
Professional	37.0	27.7
Intermediate	35.4	51.3
Routine	24.3	18.5
Missing	3.3	2.5
Educational level, %		
Higher	40.0	50.1
Middle	46.3	42.6
Lower	13.6	7.3
Missing	0.1	0.1
Job strain, %		
No strain	84.3	78.8
Strain	15.7	21.2

**Figure 2.**
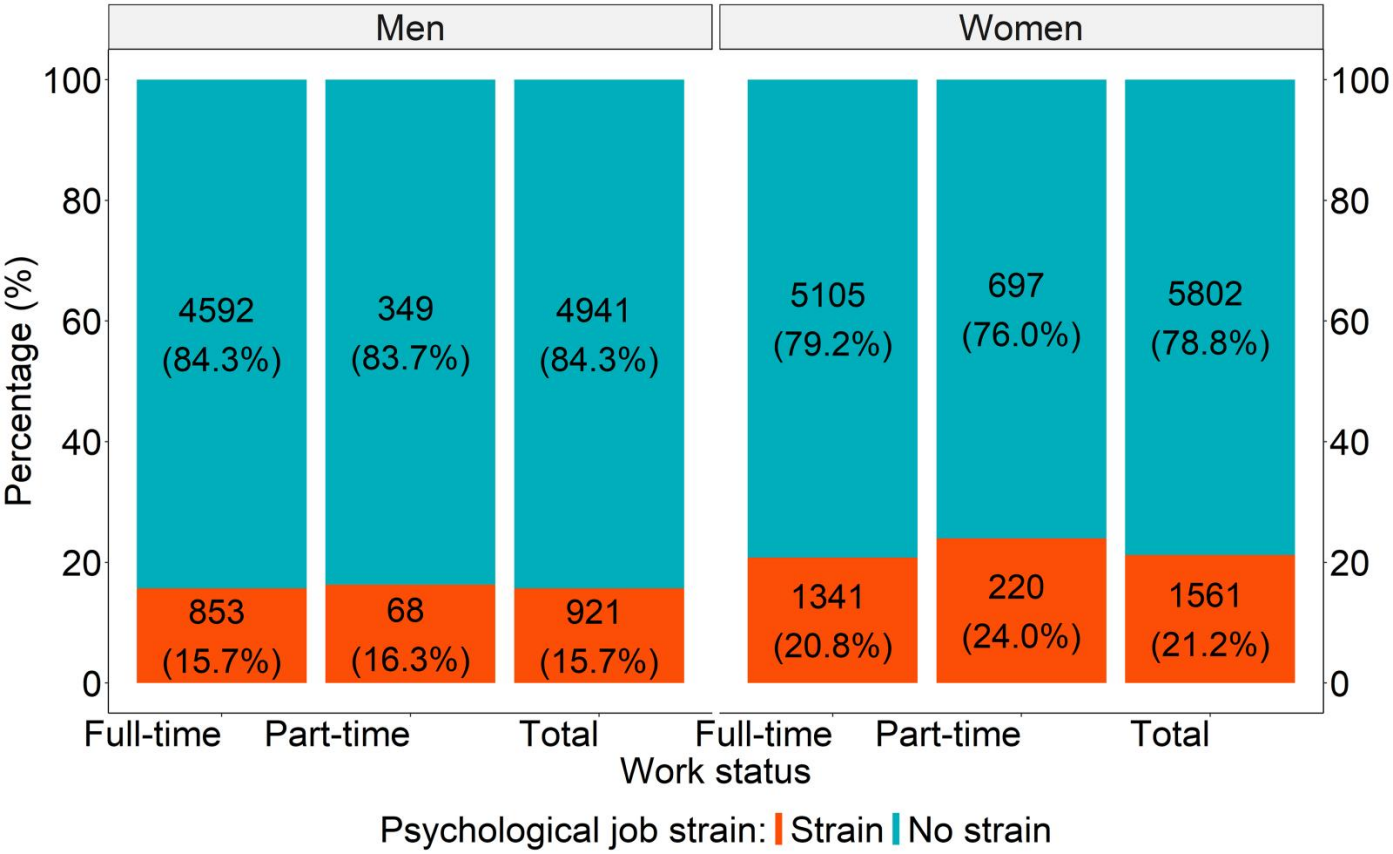
Percentage distribution of job strain and, work status for men and women.

Figure 2 shows the percentage distribution of job strain among individuals ages 50 years and older at baseline (2008–2018) among those working full-time and part-time. The left panel shows the percentage distribution for men; the right panel for women. Job strain is lower for men both in full-time and, part-time compared to women. Among men, 15.7% of older workers in full-time jobs, 16.3% in part-time jobs, and 15.7% in both full and part-time (total) jobs were exposed to job strain. Among women, 20.8% of older workers in full-time jobs, 24.0% in part-time jobs, and 21.2% in full and part-time (total) jobs were exposed to job strain. Notably, women in part-time jobs, experienced job strain more often than men.

We estimated WLE based on the multistate models illustrated in Fig. 1. The first model estimates total WLE by job strain while adjusting for sex. The second model estimates full and part-time WLE by job strain, adjusting for. We also estimated these models separately by job strain while adjusting for sex, education, and occupation based on prior evidence that suggests different levels of WLE may be observed depending on the measure of socioeconomic status used [37]. The multistate model showed a significant association between transition intensities and job strain (Supplementary Tables S1 and S2). The estimates of WLE and corresponding 95% confidence intervals are shown in Table 2. The figures included total, full-time, and part-time WLE.

**Table 2.** Total, full-time, and part-time working life expectancies at age 50 years by job strain and, sex

	WLE (95%CI)		
Covariates/exposure	Total^a^	Full-time^b^	Part-time^b^
*Men*			
No strain	13.44 (13.24, 13.65)	11.68 (11.49, 11.84)	1.76 (1.63, 1.90)
Strain	12.72 (12.34, 12.99)	11.13 (10.79, 11.37)	1.60 (1.39, 1.81)
*Women*			
No strain	12.82 (12.64, 12.97)	11.00 (10.84, 11.13)	2.00 (1.87, 2.13)
Strain	12.16 (11.86, 12.43)	10.50 (10.24, 10.72)	1.91 (1.67, 2.13)

a Estimates are from the three-state model.

b Estimates are from the four-state model.

Focusing on Table 2, the results show that men generally have slightly higher WLE estimates than women. The results suggest that job strain is associated with fewer average working years from age 50 years for both men and women, regardless of full or part-time jobs. For example, men with job strain are expected to work 0.55 years fewer as full-time at age 50 years compared to those with no job strain, and the difference is statistically significant (95% CI: 0.52–0.76). The trends were similar for women also. However, women at age 50 years work slightly longer years in part-time than men irrespective of whether they are exposed or not exposed to job strain. We found statistically significant differences in WLE between those exposed to job strain and those not exposed to job strain in the range of 0.50–0.72 years across sex included in the three-state models (full-time and total).

In summary, exposure to job strain is associated with working, on average, fewer years from age 50 years, net of sex, occupational class, and educational level.

## Discussion

In the current study, we found that women and men ages 50 years and older, who were exposed to high job strain, could be expected to have shorter working lives, on average, beyond the age of 50 years compared to those who were not exposed to high job strain. These differences were observed in models for the total WLE, full-time WLE, and part-time WLE, for men and women, and both indicators of SES (occupational class, and educational level). We further found that differences in WLE by occupational level were slightly larger than differences by educational level. Also, there were smaller differences in part-time WLE than full-time WLE across sex, occupation, and education. Consistent with prior research [38, 39] on work exit, our findings showed that it matters whether one is in a higher-level occupation (professional/higher) compared to a lower-level occupation (routine/lower). Professionals tend to work until later in life than routine workers.

The current study extends the literature by using WLE—a holistic summary measure of the length of a working life—to better understand how psychosocial working conditions affect long-term labor market participation [3–5, 8, 10]. Notably, this study underscores the significance of job strain among older workers regardless of sex, occupation, and educational level. We did, however, find a sex disparity in WLE with exposure to job strain, particularly among women in routine jobs, which is consistent with prior studies that have shown higher rates of job strain among women [40] and lower WLE among women in lower-status, physically demanding jobs [24].

Our finding that job strain is associated with shorter WLE adds to previous research showing that adverse working conditions are associated with shorter working lives. A study of Dutch employees [43] found that self-reported adverse psychosocial working conditions (e.g. job demands, autonomy, emotional demands, and social support at work) were associated with involuntary working years lost between ages 50 and 66 years, but did not examine job strain. Associations have also been found for physically demanding jobs [24, 41]. Methodological differences between our study and prior studies in measures of both work exposure (e.g. whether based on job exposure matrices or self-reported) and work status (e.g. whether self-reported or from register data; whether or not long-term sickness absence is classified as “not working”) make it difficult to directly compare the magnitude of our estimates of WLE for job strain with studies reporting WLE for other working conditions. It is also possible that those with high job strain are more likely to have left employment after age 50 years prior to the start of the study, potentially a so-called “healthy worker effect,” leading to smaller differences between groups. This illustrates a need for further investigations into the impact of psychosocial working conditions on WLE to better understand how working conditions might affect older people’s working life. These types of studies will also help to inform policies aimed at increasing the length of the working life.

As governments seek strategies to offset the rising costs pension and healthcare systems due to longer life expectancy, it may be worthwhile to target efforts toward improving psychosocial working conditions for all workers and especially for older workers. Our findings suggest that job strain is indeed a potentially important factor associated with how long women and men remain in paid work. Yet, it may be one of several negative psychosocial working conditions that aging workers face that may indeed counter current strategies to raise retirement ages. Several studies have identified a range of other factors that influence WLE, including depressive symptoms, physical work demands, and disability [24, 42, 43]. We extend the literature and argue that a holistic understanding of WLE requires a more thorough investigation of how a potentially complex mixture of psychosocial working conditions is associated with whether a person remains in work at older ages.

The study’s strengths lie in its longitudinal design, large-scale dataset, and the utilization of multi-state models. This modeling approach allows for a dynamic accounting of changes in working statuses over the later life cycle. This approach is more robust than the traditional reliance on average retirement age as an indicator of labor market exits, particularly in a historical context in which many older workers have diverse employment patterns like part-time work, bridge jobs, and/or rejoining the workforce after retirement.

All observational studies have limitations, and this study is no exception. First, we rely on self-reported measures of job strain, which could introduce bias associated with recall and/or social desirability, and effectively downwardly or upwardly biasing our estimates. Regarding social desirability bias, it is possible that some may under-report job demands in the interest of appearing content with their lives. Moreover, people with different health disorders may over-report job demands, and the same people might leave paid work earlier. Also, we were not able to control for a wide range of covariates (such as self-reported health or shift work) that may affect both job strain and WLE. This was due to the complexity and computational intensity of the multi-state models and the limited statistical power available in our data. Moreover, we could not include older workers who did not provide information on job strain at baseline in the analysis, which also may have also led to an underestimation of WLE for older workers without job strain. This may underlie the slightly smaller differences of WLE between older workers with and without job strain than we expected.

## Conclusion

This study demonstrates the importance of job strain in WLE among workers aged 50 years and older. The implications of these findings are that policies that aim to promote longer working lives that take psychosocial working conditions into account may have better success—a message that extends not only to policymakers, but also to employers. Such efforts might support longer working lives.

## Supplementary Material

ckae186_Supplementary_Data

## Data Availability

Given restrictions from the ethical review board and considering that sensitive personal data are involved, it is not possible to make the data freely available. Access to the data may be provided to other researchers in line with Swedish law and after consultation with the Stockholm University legal department. Requests for data, stored at the Stress Research Institute, Department of Psychology, should be sent to registrator@su.se with reference to the study or directly to the corresponding author. Key points Increased retirement ages extend work lives despite challenging working conditions for older individuals. Job strain, defined by Karasek’s model as high job demands and low job control, is a significant risk factor for poor health and early or disability retirement. Working life expectancy (WLE) measures the expected duration individuals will remain in paid work, accounting for diverse work statuses and labor market dynamics. The study finds that both women and men in Sweden exposed to job strain have shorter WLEs compared to those not exposed, regardless of socioeconomic status. The findings highlight the importance of improving psychological working conditions to potentially increase WLEs for aging workers, influencing policy-making.
