# Supplementary material for: Association between job strain and working life expectancy: a longitudinal study of older people in Sweden
Source: Eur J Public Health. 2024 Dec 12;35(1):85–90. doi: 10.1093/eurpub/ckae186 (PMC11832150; doi:10.1093/eurpub/ckae186)
Supplement: ckae186_Supplementary_Data [file ckae186_supplementary_data.docx]

**Table S1:** Total, full-time, and part-time working life expectancies at age 50 by job strain, occupational class, and sex [*N* = 51, 852 persons-observations].

|  | **Working life expectancy (95%CI)** | | |
| --- | --- | --- | --- |
| **Covariates/exposure** | **Total***^*^* | **Full-time***^†^* | **Part-time***^†^* |
| *Men* |  |  |  |
| Professional |  |  |  |
| No strain | 13.88 (13.60, 14.18) | 12.03 (11.75, 12.27) | 1.74 (1.56, 1.92) |
| Strain | 13.20 (12.78, 13.69) | 11.59 (11.26, 11.95) | 1.54 (1.28, 1.78) |
| Intermediate |  |  |  |
| No strain | 13.22 (12.98, 13.48) | 11.71 (11.51, 11.95) | 1.55 (1.40, 1.70) |
| Strain | 12.56 (12.21, 12.93) | 11.20 (10.90, 11.51) | 1.36 (1.16, 1.56) |
| Routine |  |  |  |
| No strain | 13.04 (12.75, 13.30) | 11.33 (11.10, 11.55) | 1.87 (1.68, 2.10) |
| Strain | 12.39 (12.06, 12.74) | 10.91 (10.60, 11.20) | 1.72 (1.46, 1.97) |
| *Women* |  |  |  |
| Professional |  |  |  |
| No strain | 13.33 (13.05, 13.66) | 11.36 (11.13, 11.59) | 2.00 (1.79, 2.24) |
| Strain | 12.69 (12.30, 13.08) | 11.00 (10.70, 11.33) | 1.86 (1.59, 2.13) |
| Intermediate |  |  |  |
| No strain | 12.71 (12.50, 12.92) | 11.04 (10.88, 11.20) | 1.78 (1.63, 1.95) |
| Strain | 12.05 (11.74, 12.35) | 10.57 (10.30, 10.83) | 1.66 (1.47, 1.87) |
| Routine |  |  |  |
| No strain | 12.55 (12.28, 12.83) | 10.67 (10.42, 10.88) | 2.33 (2.10, 2.59) |
| Strain | 11.91 (11.57, 12.30) | 10.32 (10.02, 10.58) | 2.25 (1.99, 2.55) |

*^*^* Estimates are from the three-state model.
*^†^* Estimates are from the four-state model.

**Table S2:** Total, full-time, and part-time working life expectancies at age 50 by job strain, educational level, and sex [*N* = 52, 100 persons-observations].

|  | **Working life expectancy (95%CI)** | | |
| --- | --- | --- | --- |
| **Covariates/exposure** | **Total***^*^* | **Full-time***^†^* | **Part-time***^†^* |
| *Men* |  |  |  |
| Higher |  |  |  |
| No strain | 14.03 (13.74, 14.27) | 12.14 (11.90, 12.32) | 1.82 (1.67, 2.00) |
| Strain | 13.46 (13.04, 13.79) | 11.71 (11.32, 12.08) | 1.66 (1.38, 1.89) |
| Middle |  |  |  |
| No strain | 13.20 (12.91, 13.47) | 11.48 (11.24, 11.67) | 1.82 (1.63, 2.00) |
| Strain | 12.64 (12.21, 12.94) | 11.02 (10.65, 11.29) | 1.68 (1.41, 1.90) |
| Lower |  |  |  |
| No strain | 12.43 (11.97, 12.77) | 11.25 (11.84, 11.59) | 1.34 (1.14, 1.56) |
| Strain | 11.85 (11.34, 12.34) | 10.77 (10.28, 11.13) | 1.19 (0.93, 1.41) |
| *Women* |  |  |  |
| Higher |  |  |  |
| No strain | 13.26 (13.02, 13.45) | 11.37 (11.19, 11.51) | 1.98 (1.83, 2.12) |
| Strain | 12.73 (12.39, 13.02) | 10.97 (10.68, 11.26) | 1.87 (1.59, 2.10) |
| Middle |  |  |  |
| No strain | 12.50 (12.24, 12.70) | 10.76 (10.57, 10.96) | 2.04 (1.87, 2.23) |
| Strain | 11.98 (11.64, 12.26) | 10.33 (10.02, 10.56) | 1.98 (1.73, 2.22) |
| Lower |  |  |  |
| No strain | 11.76 (11.33, 12.16) | 10.56 (10.16, 10.91) | 1.53 (1.27, 1.81) |
| Strain | 11.23 (10.71, 11.67) | 10.12 (9.71, 10.46) | 1.44 (1.09, 1.70) |

*^*^* Estimates are from the three-state model.
*^†^* Estimates are from the four-state model.

**Table S3.** Estimated response proportions and number of respondents for the Labor Force Survey (LFS) and the Swedish Work Environment Survey (SWES): 2003-2011

| LFS/SWES data collection | Response proportion LFS (%)^a^ | Response proportion additional interview (%)^a^ | Response proportion SWES survey (%)^a^ | Respondents SWES^b^ |
| --- | --- | --- | --- | --- |
| 2003 | 84.0 | 86.0 | 78.0 | 9214 |
| 2005 | 82.0 | 87.0 | 75.0 | 9703 |
| 2007 | 81.0 | 88.0 | 76.0 | 7729 |
| 2009 | 80.0 | 83.0 | 75.0 | 6354 |
| 2011 | 74.0 | 80.0 | 70.0 | 7926 |

^a^ according to The Swedish Work Environment Authority.

^b^ some individuals participated in several of these cross-sectional surveys.

**Table S4.** Number of unique individuals in the SLOSH cohort, response proportions and number of respondents to the SLOSH questionnaires: 2006-2020

| SLOSH data collection | Total in the cohort | People contacted^a^ | Response proportion (%) | Respondents | Respondents in paid work^b^ |
| --- | --- | --- | --- | --- | --- |
| 2006 | 9214^c^ | 9149 | 65 | 5985 | 5141 |
| 2008 | 18917^d^ | 18734 | 61 | 11441 | 9756 |
| 2010 | 21489^e^ | 20291 | 57 | 11525 | 9132 |
| 2012 | 18917^d^ | 17409 | 57 | 9880 | 7325 |
| 2014 | 40877^f^ | 38657 | 53 | 20316 | 15358 |
| 2016 | 40877 | 38012 | 51 | 19360 | 13572 |
| 2018 | 40877 | 37043 | 48 | 17841 | 11552 |
| 2020 | 40877 | 35700 | 49 | 17489 | 10294 |

^a^ excludes people who emigrated, died, actively opted out from the study, or had unknown address at the time

^b^ in paid work at least 30% of full time during the past 3 months

^c^ including respondents from SWES 2003

^d^ including respondents from SWES 2003 and SWES 2005

^e^ including respondents from SWES 2003 and SWES 2005, plus respondents from SWES 2007 from the Stockholm and Gothenburg counties

^f^ including respondents from SWES 2003, SWES 2005, SWES 2007, SWES 2009 and SWES 2011

**Reference**

Statistics Sweden. Swedish Work Environment Research 2011. Report No. 2012:4 ENG. Swedish Work Environment Authority, Government of Sweden; 2012.

**References (additional)**

41 Chungkham HS, Ingre M, Karasek R, Westerlund H, Theorell T. Factor structure and longitudinal measurement invariance of the demand control support model: an evidence from the Swedish Longitudinal Occupational Survey of Health (SLOSH). *PLoS One* 2013; 8(8): 1-12.

42 Solovieva S, de Wind A, Undem K, Dudel C, Mehlum IS, van den Heuvel SG, Robroek SJ, Leinonen T. Socioeconomic differences in working life expectancy: a scoping review. *BMC Pub Health* 2024; 24(1):735-52.

43 Schram JL, Schuring M, Hengel KM, Burdorf A, Robroek SJ. The influence of chronic diseases and poor working conditions in working life expectancy across educational levels among older employees in the Netherlands. *Scandinavian Journal of Work, Environment & Health* 2022; 48(5): 391-400.
